# A Dynamic Multi-Scale Network for EEG Signal Classification

**DOI:** 10.3389/fnins.2020.578255

**Published:** 2021-01-13

**Authors:** Guokai Zhang, Jihao Luo, Letong Han, Zhuyin Lu, Rong Hua, Jianqing Chen, Wenliang Che

**Affiliations:** ^1^School of Optical-Electrical and Computer Engineering, University of Shanghai for Science and Technology, Shanghai, China; ^2^School of Software Engineering, Tongji University, Shanghai, China; ^3^College of Computer Science and Engineering, Shandong University of Science and Technology, Qingdao, China; ^4^Department of Otolaryngology, Head & Neck Surgery, Shanghai Ninth People's Hospital, Affiliated to Shanghai Jiaotong University School of Medicine, Shanghai, China; ^5^Department of Cardiology, Shanghai Tenth People's Hospital, Tongji University School of Medicine, Shanghai, China

**Keywords:** brain-computer interface, electroencephalography, multi-scale, Fourier transform, dynamic learning

## Abstract

Accurate and automatic classification of the speech imagery electroencephalography (EEG) signals from a Brain-Computer Interface (BCI) system is highly demanded in clinical diagnosis. The key factor in designing an automatic classification system is to extract essential features from the original input; though many methods have achieved great success in this domain, they may fail to process the multi-scale representations from different receptive fields and thus hinder the model from achieving a higher performance. To address this challenge, in this paper, we propose a novel dynamic multi-scale network to achieve the EEG signal classification. The whole classification network is based on ResNet, and the input signal first encodes the features by the Short-time Fourier Transform (STFT); then, to further improve the multi-scale feature extraction ability, we incorporate a dynamic multi-scale (DMS) layer, which allows the network to learn multi-scale features from different receptive fields at a more granular level. To validate the effectiveness of our designed network, we conduct extensive experiments on public dataset III of BCI competition II, and the experimental results demonstrate that our proposed dynamic multi-scale network could achieve promising classification performance in this task.

## 1. Introduction

The brain sends brainwaves (Shahid et al., 2010) that enable human beings to think and act. During this process, people's motion intention can be captured by collecting EEG signals [called motor imagery (MI) EEG] from the cerebral cortex (Schlögl et al., 2005). To make MI possible, the BCI system creates a pathway between the brain and external devices (Zich et al., 2015) and converts the EEG signals into electrical signals to control peripheral devices, such as an electrically propelled wheelchair. For people suffering from physical inconveniences caused by paralysis or stroke, BCI system can help them act autonomously; this can not only help patients achieve self-care but also be a means of rehabilitation therapy (Schlögl et al., 2005; Padfield et al., 2019).

The EEG-based BCI system is divided into BCI based on steady-state visual evoked potential (SSVEP) and that based on sensorimotor rhythm (SMR) according to the type of EEG signals, and the latter is related to MI (Schlögl et al., 2005; Zich et al., 2015). The imagination of body movements affects the rhythmic activity recorded in the sensorimotor cortex. For example, when subjects are imagining movement to the left, the amplitude of mu and beta rhythm decreases on the right side of the sensorimotor areas of the brain (Shahid et al., 2010). These increases and decreases in sensorimotor rhythms are called event-related synchronization (ERS) and event-related desynchronization (ERD) respectively (Shahid et al., 2010; Padfield et al., 2019). By analyzing the characteristics of these signals and rhythms, these features can be converted into output instructions for the control of BCI system.

Focusing on the state-of-the-art MI-based EEG systems, most of them consist of two parts: feature extraction and classification (Dose et al., 2018; Padfield et al., 2019). Some systems divide the first part into feature extraction and feature selection (Bashivan et al., 2015; Schirrmeister et al., 2017; Tang et al., 2017). In the feature extraction part, informative and non-redundant features are extracted from the original EEG data. Useful features are then sent to the feature selection step to obtain less computation complexity and higher classification accuracy. Finally, the classification step matches the characteristics of the EEG signals to different categories.

For the feature extraction part, the most basic techniques are divided into time-domain, frequency-domain, and spatial domain analysis (Padfield et al., 2019). As a typical time-domain approach, autoregressive (AR) modeling used the AR coefficients or spectrum as signal features (Krusienski et al., 2006). Though it has been improved into vector autoregressive (VAR) modeling, this method was not always effective when encountering an unstable sequence (Haboub et al., 2020). As for the frequency-domain analysis, the Fast Fourier transform (FFT) and Welch's method were both widely used in this field (Oikonomou et al., 2017; Li et al., 2020). Compared to FFT, Welch's method reduced the noise information of the original data but offered lower frequency resolution. Besides, time-frequency analysis methods such as the Short-time Fourier Transform (STFT), the discrete wavelet transform (DWT), and the flexible analytic wavelet transform (FAWT) were more powerful because they related the spectral information to the temporal domain and derived dynamic features, but they also required manual screening at the same time (Kumar et al., 2014; Tabar and Halici, 2016; You et al., 2020). In terms of spatial domain analysis, common spatial pattern (CSP) was the most common method thar uses spatial filters to transform EEG signals into a new space to precisely extract useful information from different frequency bands. However, CSP was time consuming since the optimal frequency band was subject specific and had to process redundant data to find the final solution (Lotte and Guan, 2010; Yang et al., 2015; Wankar et al., 2017).

Classification methods such as support vector machine (SVM), linear discriminant analysis (LDA), Bayesian classifiers, k-nearest neighbors (k-NN), and regression trees were widely used in recent literature (Kumar et al., 2017; Oikonomou et al., 2017). Among these techniques, the LDA and the SVM approaches both had the problem of overfitting, and k-NN was memory consuming since it had to process all the datasets at once. Beyond that, logistic regression outperformed SVM, k-NN, and artificial neural network (ANNs) approaches in classification accuracy.

In addition to the classification techniques mentioned above, computational intelligence methods, including the recurrent neural network (RNN) and convolutional neural network (CNN), were also widely used (Cheng et al., 2018; Zhou et al., 2018; Tang et al., 2020). The performance of deep learning methods was compared to traditional SVM and LDA classifiers, and it was proven that neural networks can improve the classification accuracy because they can automatically select informative features and constantly adjust parameters through backpropagation (Yang et al., 2015). For example, Cheng et al. performed an experiment to improve the classification accuracy of stroke patients using deep neural networks (DNN). They found that the features selected from sub-bands by DNN outperformed traditional feature extraction methods, and the DNN classifier also performed better than SVM (Cheng et al., 2018). Except for basic CNN, a modified one-dimensional multi-scaled CNN (1DMSCNN) was proposed by Tang to classify the preprocessed EEG signals, and it proved to have a better performance compared with algorithms, including CSP and long short-term memory with Discrete Wavelet Transform (DWT-LSTM) (Tang et al., 2020). To sum up, supervised learning methods are much preferred compared to methods based on unsupervised learning, and the latter ones are mainly used for the feature selection part.

In recent years, networks like the CNN, RNN, stacked autoencoders (SAE), deep belief networks (DBN), and VGGNet (Visual Geometry Group) were widely used in MI EEG systems (Schirrmeister et al., 2017; Tang et al., 2017; Li et al., 2020). These neural networks can complete all the above-mentioned steps because the network layers can extract feature maps from original data and learn to classify according to training labels. As one of the most popular networks in MI EEG systems, the CNN was often combined with other techniques such as the WT and STFT in practical experiments. For instance, (Li et al., 2020) segmented the EEG data by time windows then employed FFT to transform each time window to spectrum. By using the modified VGG called mVGG, a complicated image containing time-frequency features was generated, and its accuracy reached 88.62, 92.28, and 96.86% on three datasets-higher than that of the state-of-the-art imaging methods (Li et al., 2020). In addition, Chaudhary et al. introduced STFT and continuous wavelet transform (CWT) into CNN and drew the conclusion that the CWT approach yields better results than the other existing methods with accuracy score of 99.35% (Chaudhary et al., 2019).

While those previous works have achieved satisfying performance on the EEG classification task, they may be limited to the ignorance of extracting the multi-scale features from different receptive fields and resolutions, and those could be an important factor in learning the contextual characteristic of the EEG signal. To handle this problem, in this paper, we propose a dynamic multi-scale network for the EEG signal classification. The proposed method is mainly based on ResNet; before we input the EEG signal to the network, we first encoded it by STFT to obtain the feature representations and decrease the influence of the noise. Moreover, to extract the multi-scale and contextual characteristic from the input signal, a novel dynamic multi-scale (DMS) layer was designed as one part of the network. Finally, we conducted extensive experiments on public dataset III of BCI competition II to validate the effectiveness of our proposed method, and the experimental results demonstrate that our method could achieve promising results compared with other ones.

The rest of this paper is organized as follows. Section 2 describes the experimental data, the preprocessing procedure, as well as the proposed network architecture. Section 3 then introduces the evaluation metrics and presents the experiment results of different channels and network architectures. Finally, the overall conclusion of this paper is summarized in section 4.

## 2. Methodology

### 2.1. Data Description

**Figure 1:**
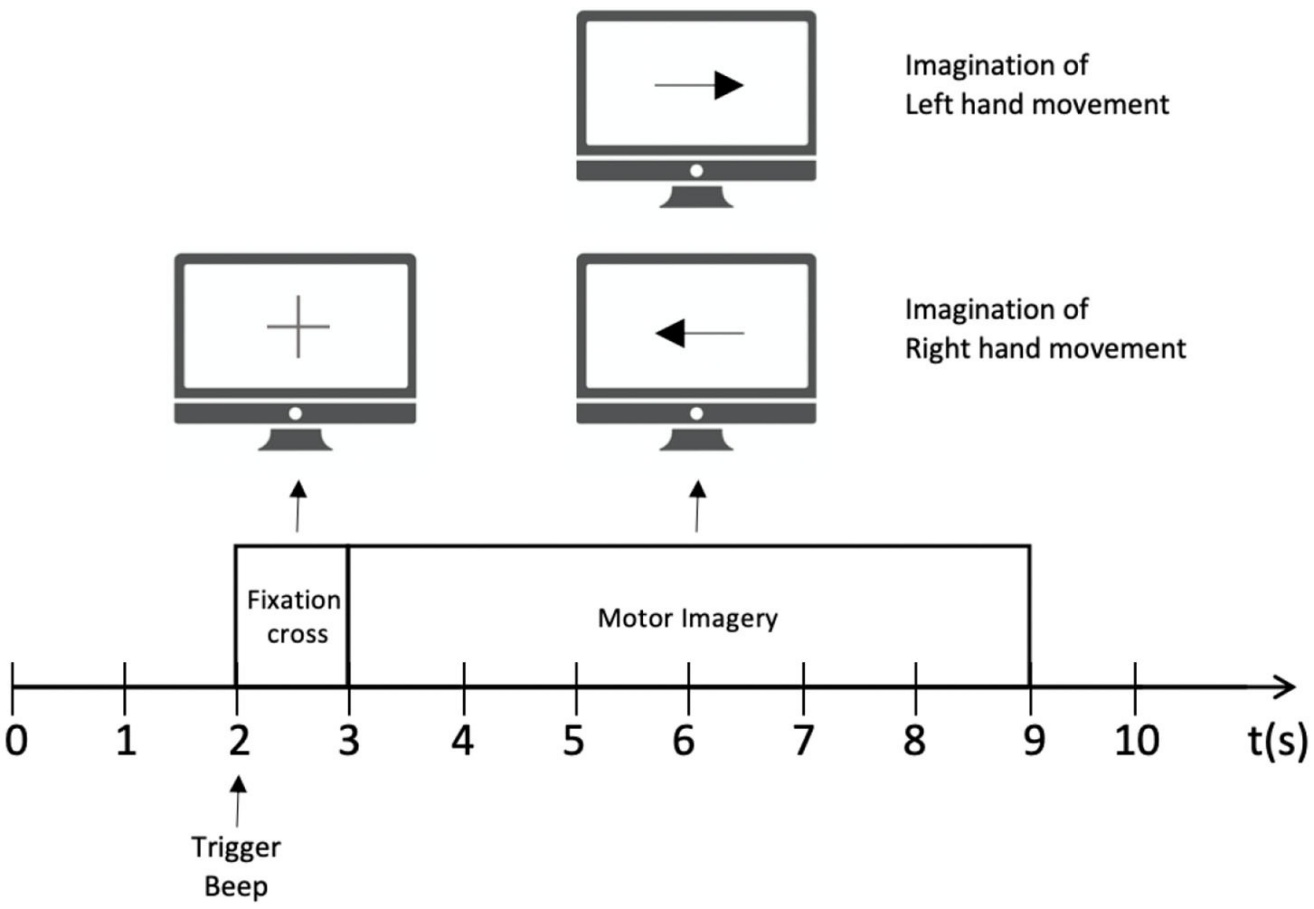
The experimental paradigm for each trial.

**Figure 2:**
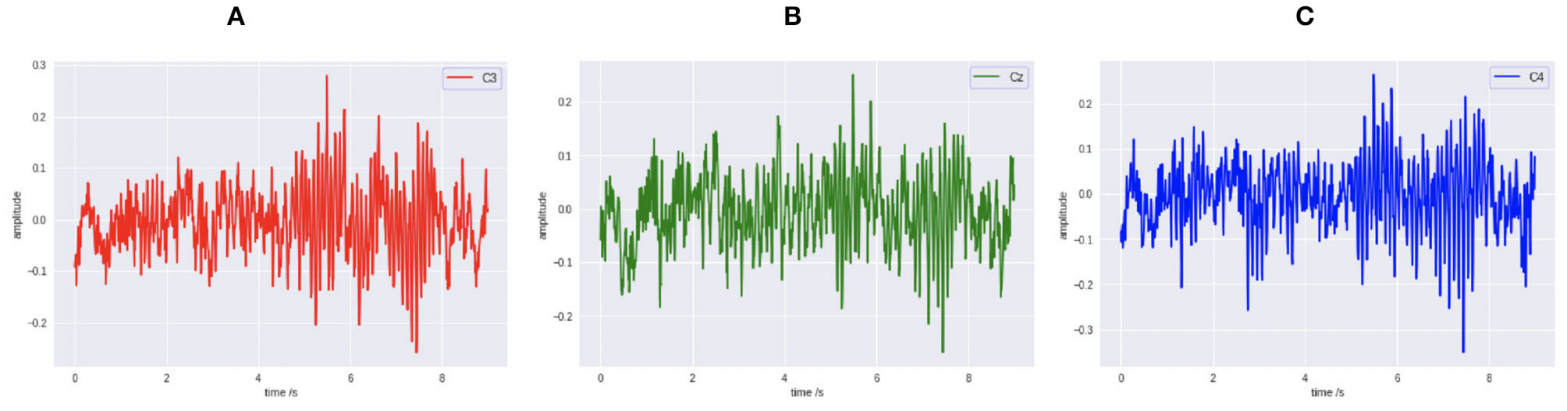
The original EEG signal data diagram. **(A)** C3. **(B)** Cz. **(C)** C4.

Public dataset III of BCI competition II is adopted to train the MI BCI model. This dataset was collected from a 25-year-old female subjects during a feedback session. This experiment is constitutive of 280 trials in total, and each trial has a length of 9 s. As shown in Figure 1, the first 2 s of the experiment was quiet. An acoustic stimulus and a cross “+,” which indicates the beginning of the trial, was then displayed in the following 1s. After that, at *t* = 3 s, an arrow (left or right, randomly) was shown on the screen as a cue. At the same time, the subject was asked to finish the motor imagery task according to the cue. The trial data were collected by three EEG channels C3, Cz, and C4, which were sampled with 128 Hz and filtered between 0.5 and 30 Hz. The diagram of source EEG data is shown in Figure 2.

### 2.2. Network Architecture

**Figure 3:**
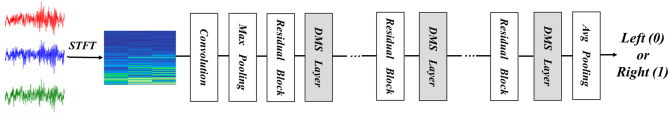
The overview of our proposed dynamic multi-scale network.

The main backbone of our proposed network is based on ResNet, which has proven its effectiveness in many computer-vision tasks. Given an input signal, we first used the STFT to gain the feature representations of the input signal and achieving the goal of noise reduction simultaneously. Furthermore, to better improve the ability of learning multi-scale features of the network, we incorporated a DMS layer after each residual block stage, which enables the network to learn the multi-scale features from the granular level. The overall structure of our designed network is shown in Figure 3, and it is sequentially composed of a series of convolution layers, max-pooling layers, residual blocks, and DMS layers. Note that for learning more non-linear information from input signals, the network uses the ReLU activation function after each convolution layer, and we omit this unit in Figure 3 for simplicity. We replace the original ResNet, which adopts average-pooling as the next to last layer, with the max-pooling layer, which can provide more salient representation and thus further improve the classification performance of the network. Finally, the extracted representations from the network pass through a fully connected layer with the softmax activation function to output the prediction probabilities of the two classes (left or right). In the following subsections, we will give a detailed description of the residual block, the data prepossessed by the STFT, and the designed DMS layer.

#### 2.2.1. Residual Block

**Figure 4:**
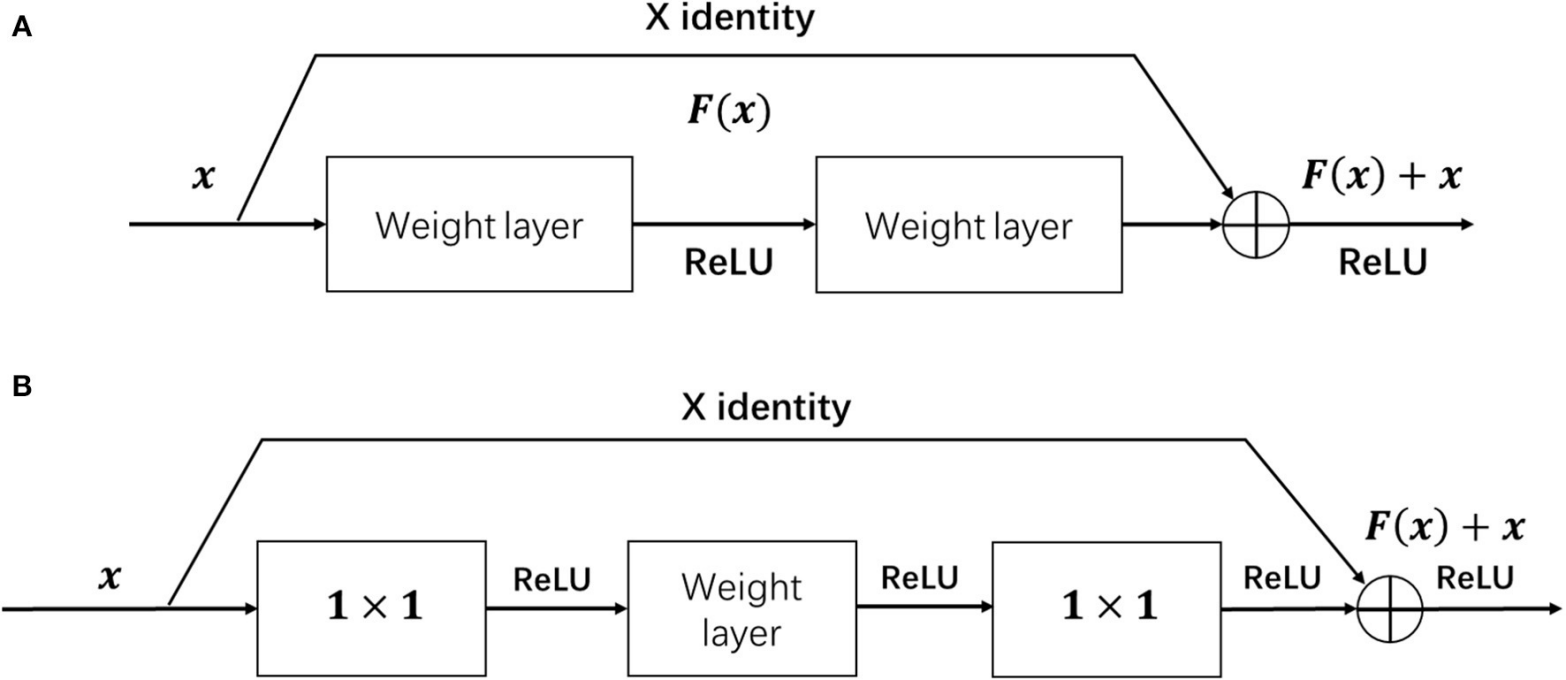
The structure of the residual block **(A)** and residual bottleneck **(B)**.

Since the whole network architecture is based on ResNet and the core unit of it is the residual block, we will in this section first give a brief retrospect of the residual block. As shown in Figure 4A, the input feature of the residual block is denoted as *x*. The residual block uses skip connection to reduce the influence of vanishing gradient problem of the network. During the process, the residual function *F*(*x*) is learned by using the labeled data to train the weight layer as shown below:

(1) $$\begin{array}{l} F\left(x\right)=F^{\prime }\left(x\right)-x \end{array}$$

where the *F*′(*x*) is the desired underlying mapping and the weight layer can be composed of any type of neural network layer, including convolutional layers or fully connected layers. Through setting the residual function *F*(*x*) to zero, the help from residual blocks to skip certain parts of the network can enable the network compose of many different feature extracting layers that capture different possible features of the data. The bottleneck architecture, as shown in Figure 4B, aims to achieve the function of controlling the dimension of feature map by adding up two 1 × 1 convolution layers before and after the weight layer.

### 2.3. Feature Encoding by STFT

Fourier Transform is a form of transforming the signal from the time domain to the frequency domain. It is an important analysis tool in the fields of acoustics, speech, telecommunications, and signal processing. In our approach, before inputting the signal into the network, we first encoded the signal by STFT. We first give a detailed description of the Discrete Fourier Transform (DFT), FFT, and STFT. DFT is a representation of continuous Fourier Transform in discrete systems, and FFT is a fast algorithm for efficiently realizing DFT. Supposing *t*(*n*) is a finite length sequence of length *N*, then the N-point DFT of *T*(*k*) is the following:

(2) $$\begin{array}{l} T\left(k\right)=\overset{N-1}{\underset{n=0}{\sum }}t\left(n\right)W_{N}^{nk},k=0,1,\ldots N-1 \end{array}$$

Among which the rotation factor *W*_*N*_ is defined as follows:

(3) $$\begin{array}{l} W_{N}=e^{-j2\pi /N} \end{array}$$

When *t*(*n*) is a complex sequence, directly calculating *T*(*k*) according to the above formula based on a certain value of *k* requires *N* complex multiplications and *N* − 1 complex numbers addition. For all *k* values, a total of *N*^2^ complex multiplications and *N*(*N* − 1) complex additions are thus required, which requires a huge workload. However the rotation factor $W_{N}=e^{-j2\pi /N}$ has its symmetrical and periodic characteristics as follows:

(4) $$\begin{array}{l} W_{N}^{k}=-W_{N}^{k+N/2} \end{array}$$

(5) $$\begin{array}{l} W_{N}^{k}=-W_{N}^{k+N} \end{array}$$

**Figure 5:**
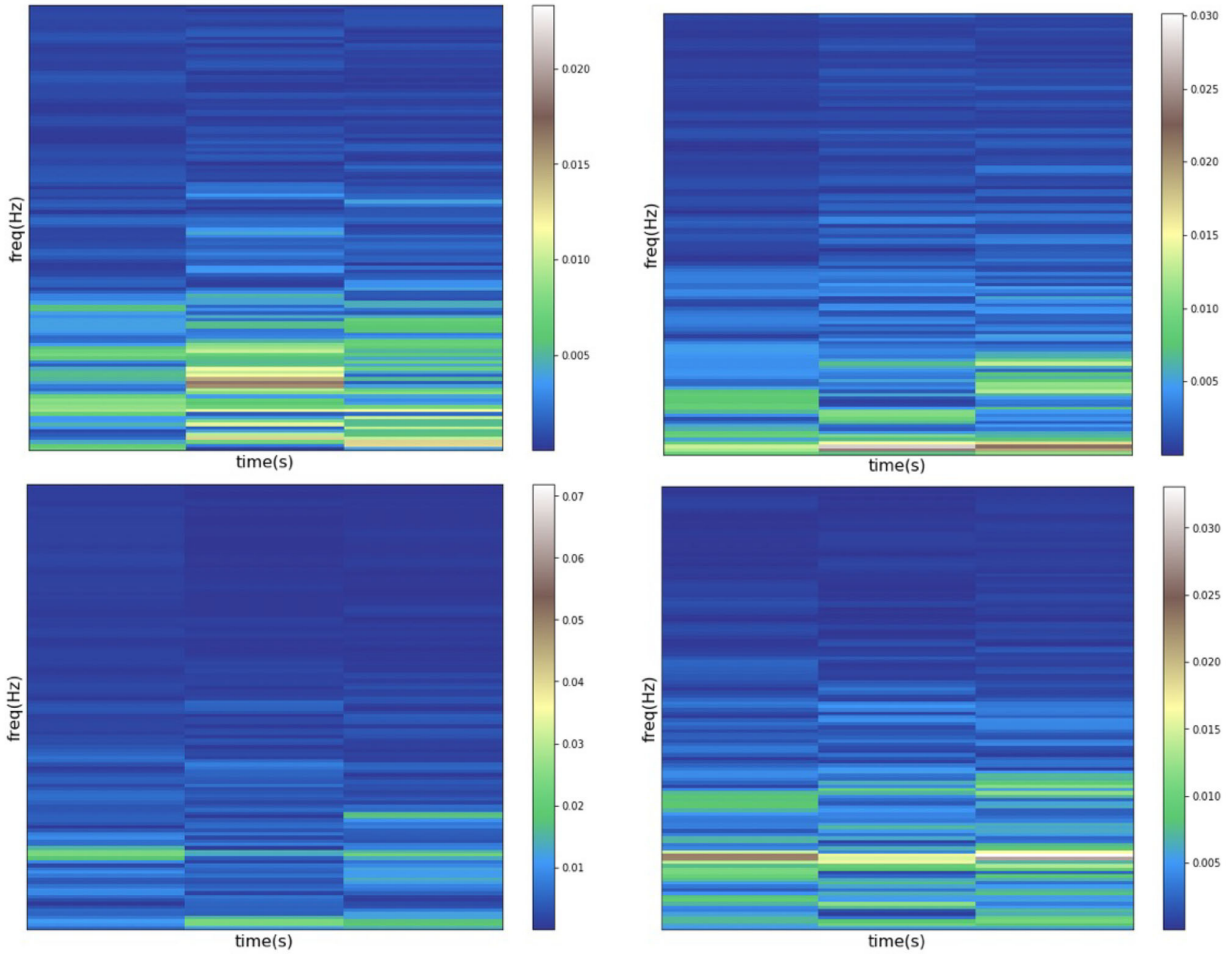
Data diagram after STFT processing.

By applying these properties, FFT decomposes the long-sequence DFT into smaller DFTs and uses these small DFT calculations to replace large DFT calculations to achieve the purpose of improving efficiency. Nevertheless, since DFT has higher requirements for sampling the entire period of the signal where non-integer sampling will cause analysis errors including spectral leakage and fence effects, the STFT is applied to solve these problems. STFT defines a time and frequency distribution class, which specifies the complex amplitude of any signal changing with time and frequency to get more accurate spectrum information. It uses a sliding window mechanism as well to set the window size and step size, allowing the window slide on the time domain signal and calculating the Fourier Transform of each window separately to form the frequency domain signal corresponding to different time windows, which is expressed as follows:

(6) $$\begin{array}{l} s_{N}\left(n\right)=s\left(n\right)g\left(n-mR\right) \end{array}$$

(7) $$\begin{array}{l} S_{STFT}\left(n,F\right)=\overset{N-1}{\underset{n=0}{\sum }}s_{N}\left(n\right)e^{-jFn}=\overset{N-1}{\underset{n=0}{\sum }}s\left(n\right)g\left(n-mR\right)e^{-jFn} \end{array}$$

among which, the signal sequence at time *n* is defined as *s*(*n*). And the *g*(*n*−*mR*) represents the selected window of size *n*−*mR*, along with the time axis *m* and the hop size of *R*. The frequency axis is defined as F. After applying STFT to the input signal, the feature representation of the data is shown as Figure 5.

### 2.4. Dynamic Multi-Scale Layer

**Figure 6:**
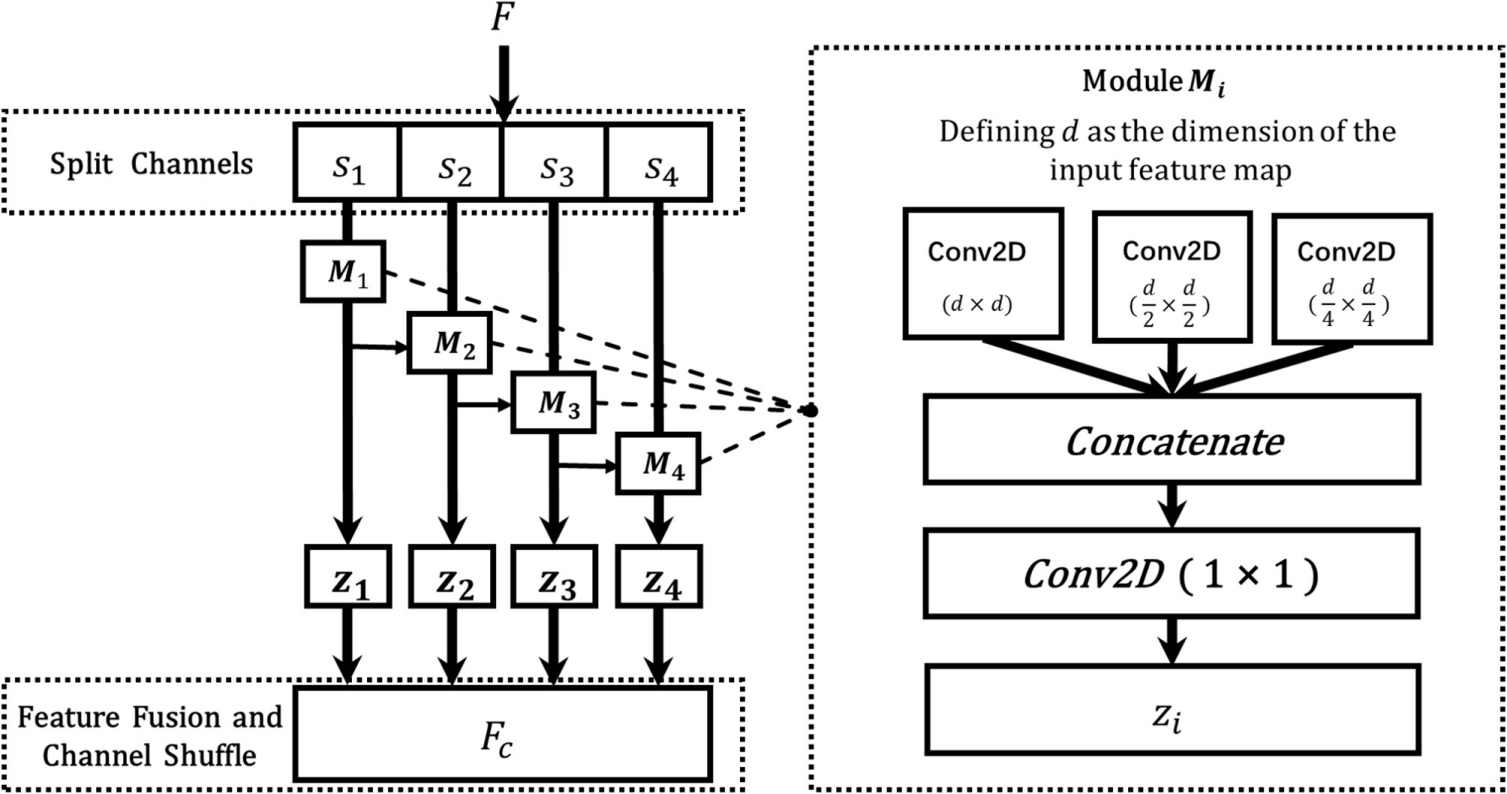
The architecture of the DMS layer. The **F** is the input feature map from the previous layer, and **s**_**1**_, **s**_**2**_, **s**_**3**_, *and***s**_**4**_ are the split feature maps from **F**, **M**_**i**_ denotes the dynamic multi-scale module, which consists of three 2D convolutions with different sizes (**d** × **d**, $\frac{d}{2}\times \frac{d}{2}$, $\frac{d}{4}\times \frac{d}{4}$), where **d** is the dimension of feature **s**_**i**_, and **z**_**i**_ is the fused output features of **M**_**i**_, and **F**_**C**_ represents the final output of the DMS layer.

The multi-scale features describe the contextual characteristics of the input from different scales, which are of great importance to the vision classification tasks. However, due to the fixed sizes of filters, the classical ResNet fails to learn the multi-scale features from different receptive fields, which hinders the model from achieving a better classification performance. To address this challenge, in this section, we designed a novel dynamic multi-scale (DMS) layer that could extract the multi-scale features more efficiently, and the structure of the DMS layer is shown in Figure 6. Given an input feature *F*, a channel split function *f* (·) is utilized to divide the feature map to four equal numbers of sub-maps, and each of them could be denoted as *s*_*i*_ where *i* ∈ {1, 2, 3, 4}. Then, to learn multi-scale features from the granular level, a dynamic multi-scale learning module *M*_*i*_ is designed as shown in the right part of Figure 6, which uses three dynamic sizes of 2D convolutions to extract the multi-scale features from different receptive fields. To balance the computational complexities and the final model performance, three sizes of *M*_*i*_ are adopted, *d* × *d*, $\frac{d}{2}\times \frac{d}{2}$, and $\frac{d}{4}\times \frac{d}{4}$, respectively, and here *d* represents the dimension of feature map *s*_*i*_. After passing through those three convolution layers, the output features are then concatenated as one. Furthermore, to reduce the numbers of the feature maps, a convolution layer with the size of 1 × 1 is utilized to output the final feature map *z*_*i*_. Specifically, inspired by the previous work (Gao et al., 2019), we add {*z*_1_, *z*_2_, *z*_3_} to {*s*_2_, *s*_3_, *s*_4_} for combining more information from different scales. After the processing from each *M*_*i*_, the learned multi-scale feature map *z*_*i*_ is gained, and the final output feature map of the DMS layer is obtained by fusing those four sub-maps *z*_1_, *z*_2_, *z*_3_, and *z*_4_ with channel shuffling. Since different feature map resolutions can contain discriminative information, and the DMS layer aims to make the network more conducive for learning multi-scale and contextual features, we located the DMS layer after each stage's last residual block for obtaining the multi-scale features more efficiently. The algorithm of dynamic multi-scale feature learning process is illustrated in Algorithm 1.

**Algorithm 1:**
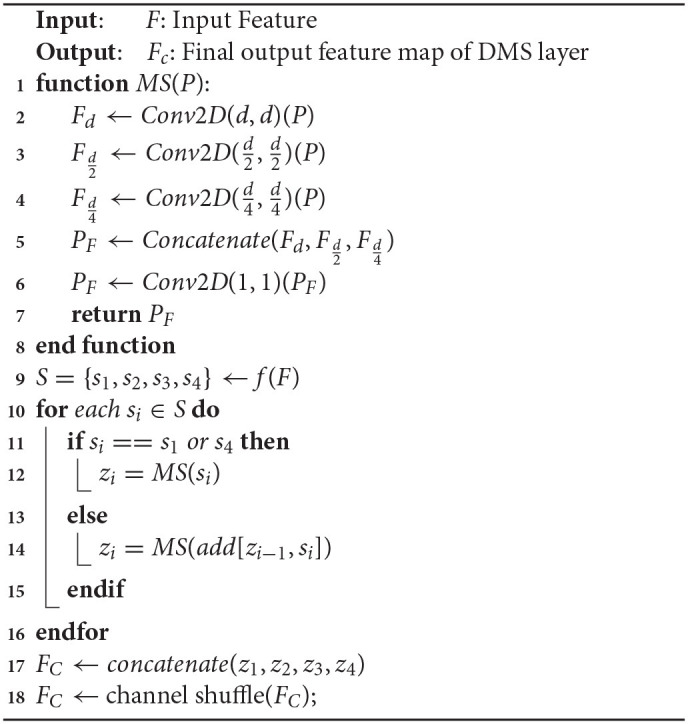
The algorithm of dynamic multi-scale feature learning

### 2.5. Implementation Details

The experiment runs on Nvidia GTX1080 GPU and is implemented by Keras 2.2.5. The categorical cross-entropy loss function is adopted to train the CNN model, which assesses the difference between the real label and the predicted label. As for the network optimizer, the Adam optimizer was chosen to adaptively optimize the learning rate based on the initial setting of 0.0003. Except for that, we also use the callback function ReduceLROnPlateau to monitor the decline in learning rate according to validation accuracy, and the lower boundary of learning rate was then set to 0.0001 and the patience set to 10 epochs. The training set and the validation set were divided according to the scale of 0.3, and the former was trained with a batch size of 8 for each epoch. By using softmax as our classifier, the checkpoint with the best accuracy was selected as the final model.

## 3. Experimental Results

### 3.1. Evaluation Metrics

For evaluation of experimental results, the commonly used accuracy metric was adopted. In this experiment, accuracy was evaluated by judging the classification results of two classes of the model, and the metric is defined as below:

(8) $$\begin{array}{l} Accuracy=\frac{TP+TN}{TP+TN+FP+FN} \end{array}$$

where TP (True Positives) refers to the number of the EEG records that indicate left and identified as left; TN (True Negatives) denotes the number of the EEG records that are left and identified as right; FP (False Positives) is the number of the EEG records that are right but are predicted as left; and FN (False Negatives) refers to the number of the EEG records that are right but are predicted as right. By using this metric, the performance of the proposed model could be evaluated quantitatively.

### 3.2. Performance of Different Channels and Window Functions

In this section, an experiment of different channel combinations and STFT windows was conducted to compare the corresponding performance. The selected channel combinations included 2-channel (C3 and C4) and 3-channel (C3, Cz, and C4). STFT windows are adopted to reduce the leakage of the spectrum during signal interception. There are some widely used STFT window functions from which we adopted boxcar, triang, hamming, hann, and bartlett in this experiment.

The comparison result is shown in Table 1. It demonstrates that the best performance is achieved on the combination of 2-channel and hann window function with the accuracy of 90.47%, and the hardest classification is 3-channel boxcar function with the accuracy of 65.48%. We also notice that the overall performance of 2-channel input data is relatively better, which means that the EEG signals under the Cz region contain less informative characteristics but more noise. Meanwhile, the hann window achieves higher accuracy than other STFT window functions, which means this default Fourier function indeed has the best performance. Overall, the accuracy performance of different window combinations of 2-channel is above 77%, which indicates that the proposed network architecture is relatively effective in this classification scenario.

**Table 1:** The overall performance of different channel and window combinations.

**Method**	**Accuracy (%)**
boxcar + C3/C4	83.33
triang + C3/C4	89.28
hamming + C3/C4	86.90
hann + C3/C4	90.47
bartlett + C3/C4	77.38
boxcar + C3/Cz/C4	65.48
triang + C3/Cz/C4	70.24
hamming + C3/Cz/C4	67.86
hann + C3/Cz/C4	66.67
bartlett + C3/Cz/C4	77.35

### 3.3. Comparison With Different Sampling Intervals

As the experimental paradigm shown in Figure 1, the cue of the arrow appears after 3 s, and the motor imagery begins directly after. The informative EEG signals from 3 to 9 s are therefore adopted as input data in this paper. However, the signals of the whole period of 6 s contains unrelated noise. Meanwhile, considering the delay between the time when the subject receives the cue and when she starts to imagine, the sliding window of the time duration is introduced to bring as little irrelevant noise as possible into this experiment. As shown in Table 2, the durations of each 3 s after the first 3 s are selected as input data, and the comparison results show that the classification accuracy between the period of 3–6 s is the best. Correspondingly, the accuracy based on the 5–8 s section performs the worst, which suggests that there is indeed a short delay before the subject conduct the motor imagery after receiving the cue. Comparing the overall results of different time durations, the accuracy decreases together with time interval is all above 78%, and it can thus be considered that motion imagination mainly occurs in a short period after receiving the cue. According to this part of the experiment, we find that the classification accuracy is indeed related to the time interval sliding window. However, due to the differences of delay time in each subject, specific experiments and analyses need to be conducted.

**Table 2:** The overall performance of different sampling intervals.

**Duration (s)**	**Accuracy (%)**
3–6	90.47
3.5–6.5	86.90
4–7	84.52
4.5–7.5	79.76
5–8	78.57

### 3.4. Comparison With Different Combinations of Convolutions

Different combinations of convolutions in the DMS layer could give various representations from different receptive fields. Thus, in this section, we conduct the experiments to explore the effectiveness of different combinations of convolutions. Since we have adopted three sizes of convolutions in DMS layer, here we denote Conv_1_, Conv_2_, and Conv_3_ as the convolution with the sizes *d* × *d*, $\frac{d}{2}\times \frac{d}{2}$, and $\frac{d}{4}\times \frac{d}{4}$, respectively, where *d* is the dimension of the input feature map from the previous layer. The detailed comparison result is shown in Table 3, from the result we can see that the best performance is gained by the combination of Conv_1_, Conv_2_, and Conv_3_, simultaneously, which with the accuracy of 90.47%. Meanwhile, for single size of the convolution, the best result is achieved by Conv_2_, which indicates that the medium size of the convolution can be crucial in this classification task.

**Table 3:** The overall performance of different combinations of convolutions.

**Method**	**Accuracy (%)**
Conv_1_	86.97
Conv_2_	88.21
Conv_3_	87.32
Conv_1_ + Conv_2_	89.73
Conv_1_ + Conv_3_	89.22
Conv_2_ + Conv_3_	89.65
Conv_1_ + Conv_2_ + Conv_3_	90.47

### 3.5. The Effectiveness of Different Split Channel Numbers

In our proposed method, different split numbers of the feature channels could provide various influences on the final result. Therefore, in this section, we implement 1 to 6 split-channel numbers to explore its effects on the final classification performance. As illustrated in Table 5, with the split-channel number increasing, the performance of the classification model is improved. Specifically, when the split-channel number is more than 4, the boosted performance is not as comparative as the previous ones. Thus, considering to balance the model performance and complexities, we adopt the split channel number of 4 as our final experimental setting.

### 3.6. Compare With Other Methods

To further evaluate the effectiveness of our proposed network, we compared our method with other previous works, including STFT based features+ResNet (He et al., 2016), STFT based features+CNN (Li et al., 2014), STFT based features+Res2Net (Gao et al., 2019), PSD+LDA (Solhjoo and Moradi, 2004), Discriminative area selection+FHN (Hsu, 2015), DWT and AR model+LDA (Xu et al., 2009), Wavelet based features+FSVM/SVM/CMM (Xu et al., 2019), Multiple auto correlation+LVQ (Wang et al., 2014), Morlet wavelet+Bayes quadratic (Lemm et al., 2004), Higher order features+LDA/Neural network (Zhou et al., 2008). Table 4 shows the comparison results of networks above. According to the classification accuracy demonstrated in Table 4, it can be observed that the proposed network performs better in this EEG classification task than the other two ones. The best performance of our method can achieve the accuracy of 90.47% since we adopt STFT for preprocessing and incorporate the DMS layer to our network. In conclusion, it is proved that with the adoption of DMS layer, our proposed method can achieve a promising performance compared to other common networks.

**Table 4:** The overall performance of different methods.

**Method**	**Accuracy (%)**
PSD+LDA	65.60
Discriminative area selection+FHNN	83.10
STFT based features+ResNet	85.09
STFT based features+Res2Net	89.73
DWT and AR model+LDA	90.00
Wavelet based features+FSVM	87.86
Wavelet based features+SVM	89.83
Wavelet based features+CNN	84.09
Multiple auto correlation+LVQ	90.00
Morlet wavelet+Bayes quadratic	89.29
Higher order features+LDA	89.29
Higher order features+Neural network	90.00
STFT based features+CNN	80.95
Our Proposed Method	90.47

**Table 5:** The overall performance of different split channel numbers.

**Split Channel Numbers**	**Accuracy (%)**
Ch-1	87.61
Ch-2	88.53
Ch-3	89.79
Ch-4	90.47
Ch-5	90.50
Ch-6	90.51

## 4. Conclusion

In this paper, we propose a dynamic multi-scale network for the motor imagery EEG signals classification, which could help patients achieve self-care and rehabilitation therapy potentially. The main backbone of the proposed network is based on ResNet, and, given input from the network, we first encoded the feature representations by STFT; to further learn the multi-scale features from a more granular level, the proposed network incorporates a dynamic multi-scale layer that enables us to learn more contextual information from different receptive fields. To evaluate the performance of our proposed method, we conducted extensive experiments on public dataset III of BCI competition II. The experimental results demonstrate that our proposed method could achieve a competitive result, which further proves the effectiveness of the designed network. In future work, we will focus on exploring the combination of pre-defined features with the deep convolution features.

## Data Availability Statement

Publicly available datasets were analyzed in this study. This data can be found here: http://www.bbci.de/competition/ii/.

## Author Contributions

GZ, JL, and LH conceived the idea and designed the algorithm. GZ and JL conducted the experiments and validated its effectiveness. LH wrote the initial paper. All authors contributed to refining the ideas and revised the manuscript.

## Conflict of Interest

The authors declare that the research was conducted in the absence of any commercial or financial relationships that could be construed as a potential conflict of interest.
